# Expanding the Scope of Interventional Oncology: Locoregional Therapies in Extrahepatic Malignancies

**DOI:** 10.3390/cancers17050726

**Published:** 2025-02-21

**Authors:** Gavin Wu, Cindy Chen, Jin Chang, Farbod Fazlollahi, Mina S. Makary

**Affiliations:** Department of Radiology, The Ohio State University Wexner Medical Center, Columbus, OH 43210, USA; gavin.wu@osumc.edu (G.W.); cindy.chen@osumc.edu (C.C.); jin.chang@osumc.edu (J.C.);

**Keywords:** interventional oncological treatments, combination treatments, transarterial chemoembolization, transarterial chemoperfusion, transarterial embolization, transarterial radioembolization, selective internal radiotherapy, locoregional therapies, extrahepatic cancers, glioblastoma, soft tissue sarcoma, pancreatic cancer, prostate cancer, renal cell carcinoma, tumor control, palliative care, minimally invasive oncology

## Abstract

Background/Objectives: Locoregional therapies (LRTs), including transarterial embolization (TAE), transarterial chemoembolization (TACE), and transarterial radioembolization (TARE), have become integral in the management of hepatocellular carcinoma (HCC) in recent decades and continue to shape evolving treatment strategies. While their role in liver tumor management is well established, their potential for treating extrahepatic malignancies is gaining increasing attention. Notably, growing research has highlighted the promising applications of TAE, TACE, and TARE in extrahepatic cancers such as glioblastoma (GBM), soft tissue sarcomas (STSs), prostate cancer (PCa), pancreatic cancer, and renal cell carcinoma (RCC). This review aims to explore these novel applications, providing a comprehensive summary of the current literature, examining clinical outcomes, and discussing future directions for integrating these techniques into broader oncologic treatment strategies. Methods: A systematic literature review was conducted focusing on LRTs such as TAE, TACE, and TARE in extrahepatic malignancies. Studies published between May 1998 and December 2024 were included, emphasizing outcomes in GBM, STS, PCa, pancreatic cancer, and RCC. Data extraction prioritized clinical outcomes, safety profiles, and procedural efficacy. Results: LRTs demonstrated significant potential in managing extrahepatic malignancies, with TAE, TACE, and TARE showing promising results in palliative management and tumor control. Across studies, these therapies exhibited varying degrees of success in improving progression-free survival and overall survival, with minimal systemic toxicity. Conclusions: The expanding application of LRTs in extrahepatic malignancies highlights their transformative potential in interventional oncology. By offering targeted, minimally invasive treatment options, these modalities bridge critical gaps in managing tumors refractory to conventional therapies. Future research should focus on standardizing protocols, optimizing patient selection, and exploring combination therapies to maximize their clinical efficacy.

## Simple Summary

Locoregional therapies, such as transarterial embolization, transarterial chemoembolization, and transarterial radioembolization, have traditionally been used to treat liver malignancies like hepatocellular carcinoma. However, more recently, their potential in treating extrahepatic cancers, such as glioblastoma, soft tissue sarcomas, prostate cancer, pancreatic cancer, and renal cell carcinoma, has gained increasing attention. This review explores the latest advancements in the utilization of these therapies for extrahepatic malignancies. Through a detailed examination of the literature to date, we highlight promising results in tumor control, progression-free survival, and symptom management, with each modality offering unique benefits. While these techniques show significant potential in expanding the treatment paradigm for certain cancers, challenges remain, including variability in protocols and long-term outcomes.

## 1. Introduction

Locoregional therapies (LRTs) have long been a pillar of interventional oncology, offering minimally invasive options that deliver targeted treatment directly to tumors while minimizing systemic toxicities and complications. Developed in the 1960s and 1970s, these therapies were initially used to manage unresectable liver tumors, such as hepatocellular carcinoma (HCC). By delivering therapeutics to the tumor vasculature, LRTs introduced a transformative approach in cancer treatment that has significantly advanced patient care and outcomes.

Among the most widely utilized LRTs are transarterial embolization (TAE), transarterial chemoembolization (TACE), and transarterial radioembolization (TARE). TAE relies solely on blocking the blood supply to a tumor without the addition of drugs or radiation, primarily inducing ischemia in the targeted area [1]. In contrast, TACE combines embolization with the local delivery of chemotherapeutic agents, effectively inducing ischemia in the tumor while simultaneously releasing cytotoxic agents to the affected tissue [2]. Formally introduced in clinical practice in 1977, the use of TACE quickly gained traction as early studies demonstrated improved overall survival (OS) compared to supportive care alone [2,3]. It has since become a cornerstone therapy for intermediate-stage HCC, and its efficacy in the liver has led to increasing interest in adapting TACE for other challenging cancer types.

Following TACE’s success, further innovation in LRTs led to the development of TARE, a technique that uses radioactive microspheres to deliver high-dose radiation directly to tumor cells within the liver. Initially introduced as a complementary therapy for patients with limited chemotherapy tolerance or in cases where selective radiation delivery provided distinct advantages, TARE has become a valuable option in HCC treatment, particularly for patients with portal vein invasion or those unsuitable for TACE [4,5].

Despite historically being employed in the treatment of hepatic malignancies, the potential applications of these LRTs are expanding beyond the liver. The ability to precisely target vasculature feeding malignant tissues has fueled interest in adapting LRTs for extrahepatic cancers, particularly tumors that are refractory to conventional treatments or have limited response rates to systemic therapies. For instance, the favorable outcomes of TARE in tumor control has fostered research into potential applications for aggressive and hard-to-reach tumors outside the liver, and TAE’s utility to induce ischemia is increasingly being explored in cancers where vascular supply is a critical factor in tumor growth [6,7]. Recent studies and clinical trials have yielded promising results, highlighting the potential of TAE, TACE, and TARE as potential treatments for extrahepatic malignancies such as glioblastoma, soft tissue sarcomas, prostate cancer, pancreatic cancer, and renal cell carcinoma. This review will examine recent advancements in the extrahepatic application of LRTs, discussing clinical outcomes, challenges, and future directions for integrating these techniques into a broader oncologic framework.

## 2. Methods

A comprehensive literature search was performed using PubMed, Embase, and Google Scholar to identify relevant studies published between May 1998 and December 2024. The search utilized a range of keywords to ensure thorough coverage, including “glioblastoma”, “soft tissue sarcomas”, “prostate cancer”, “pancreatic cancer”, “renal cell carcinoma”, “locoregional therapies”, “transarterial chemoembolization (TACE)”, “transarterial radioembolization (TARE)”, “transarterial embolization (TAE)”, “prostate artery embolization (PAE)”, “intra-arterial chemotherapy”, “tumor vasculature”, “progression-free survival (PFS)”, “overall survival (OS)”, “minimally invasive oncology”, and “LRT outcomes.” Additionally, bibliographies of relevant articles were manually screened to identify supplementary studies.

Inclusion criteria consisted of peer-reviewed studies that investigated LRTs such as TAE, TACE, or TARE in extrahepatic malignancies, with a focus on glioblastoma, soft tissue sarcomas, prostate cancer, pancreatic cancer, and renal cell carcinoma. Studies that provided data on clinical outcomes, safety profiles, procedural techniques, or patient selection criteria were prioritized. Conference abstracts, non-peer-reviewed articles, and publications not available in English were excluded from the review.

Following the application of inclusion and exclusion criteria, a total of 97 studies were selected by three independent reviewers for data extraction. Extracted data included study characteristics, patient demographics, procedural details, and key outcomes such as tumor response rates, symptom palliation, progression-free survival, and overall survival. Safety profiles were also analyzed to assess adverse events.

## 3. Emerging Extrahepatic Applications of LRTs

The success of TAE, TACE, and TARE in managing hepatic malignancies has paved the way for their utility in extrahepatic cancers. With their precision and favorable safety profiles, these LRTs are poised to redefine treatment paradigms and improve patient outcomes for cancers that have historically been difficult to treat [8,9]. Indeed, emerging research highlights the potential of these minimally invasive modalities in managing glioblastoma (GBM), soft tissue sarcomas (STSs), pancreatic cancer, renal cell carcinoma (RCC), and prostate cancer (PCa).

This section explores the expanding role of TAE, TACE, and TARE in treating these cancers, focusing on the rationale behind their use and the growing body of evidence supporting their safety and efficacy. Through this exploration, we aim to highlight the growing impact of these techniques in interventional oncology and their potential in advancing minimally invasive cancer care. Table 1 summarizes the indications, clinical outcomes, and complications of these LRTs, illustrating their promising applications in extrahepatic settings.

### 3.1. LRT for the Kidney

#### 3.1.1. Background

RCC is the most common primary malignancy of the kidney, accounting for approximately 85% of all renal cancers. Globally, RCC ranks among the top ten cancers in terms of incidence, with over 430,000 new cases diagnosed annually and more than 150,000 deaths attributed to the disease each year [48,49]. In the United States, the incidence of RCC has steadily increased, with projections indicating over 80,000 new cases and approximately 14,000 deaths in 2024 alone [50,51]. The disease predominantly affects individuals in their sixth and seventh decades of life, with a notable male predominance. Established risk factors include smoking, obesity, hypertension, and occupational exposures to carcinogens such as cadmium and herbicides [50,52,53].

A defining feature of RCC is its biological heterogeneity, comprising a range of histological subtypes with distinct molecular profiles and clinical behaviors. Clear cell RCC, the most prevalent subtype, accounts for over 70–80% of cases and is strongly associated with mutations in the von Hippel–Lindau gene. These mutations drive tumorigenesis by dysregulating hypoxia-inducible factors and promoting angiogenesis [49,53]. Non-clear cell subtypes, including papillary and chromophobe RCC, demonstrate unique genetic and clinical characteristics that influence prognosis and therapeutic responses [54]. RCC also exhibits a propensity for vascular invasion, often extending into the renal vein and inferior vena cava, and advanced disease frequently metastasizes to the lungs, bones, and brain [50]. Unfortunately, nearly 30% of patients present with metastatic disease at diagnosis, contributing to the historically poor prognosis associated with advanced RCC [55,56].

The management of RCC has evolved significantly in recent decades. For patients with slow-growing tumors or those who are not ideal candidates for surgery, active surveillance (AS) has emerged as a viable strategy, prioritizing quality of life (QoL) while deferring unnecessary interventions [11,49,53,57]. In localized RCC, surgical resection remains the standard of care, with partial or radical nephrectomy offering curative potential for early-stage disease [11,50,54]. Advances in systemic therapies, including immune checkpoint inhibitors, tyrosine kinase inhibitors (TKIs), and innovative combination regimens, have also significantly improved outcomes in advanced and metastatic RCC [50,52]. Despite these advancements, the rising global incidence of RCC and its complex biological features emphasize the importance of continued research to refine therapeutic strategies and develop more effective treatment options.

LRTs are emerging as promising alternatives and adjuncts to surgical intervention in the management of RCC. Catheter-directed therapies, including TACE and TARE, have gained traction as minimally invasive options for select patients. By directly targeting tumor vasculature, these minimally invasive procedures provide effective tumor control while reducing perioperative complications and facilitating faster recovery [57,58,59]. Combination therapies incorporating LRTs, such as embolization followed by ablation, have also demonstrated considerable promise in patients with advanced or metastatic disease [17]. While long-term efficacy data are limited, these findings demonstrate the growing role of LRTs in addressing the limitations of traditional therapies and establishing themselves as integral components of comprehensive RCC management.

#### 3.1.2. TACE

##### Outcomes and Complications

While TACE is widely utilized for treating liver malignancies, its application in RCC remains relatively limited [10]. However, recent studies have shown promising outcomes, demonstrating its potential for effective disease control in unresectable RCC. In an investigation of doxorubicin-loaded DEB-TACE, Bi et al. reported disease control rates (DCRs) of 94.1% at one month and 84.6% at six months, with a median PFS of 21.4 months and OS of 24.6 months. Similarly, a randomized trial by Karalli et al. comparing DEB-TACE to TAE demonstrated superior tumor necrosis rates with TACE, achieving an average necrosis of 88.3% on CT compared to 29.4% with TAE. Histopathologic evaluation confirmed similar results, with necrosis rates of 87.5% for TACE and 26% for TAE. This enhanced cytoreductive efficacy may be attributable to the addition of doxorubicin to the embolic agent and suggests a potential advantage of TACE over traditional embolization techniques [11].

In terms of safety, TACE has been shown to be well tolerated, with a low incidence of severe adverse events. For instance, Bi et al. reported no perioperative deaths or treatment-related adverse events of grade 3 or higher. However, mild complications such as abdominal pain, nausea, and vomiting were noted. Other minor issues included moderate fever and a single case of grade 1 hematuria, both of which were successfully managed with supportive care [10]. Similarly, Karalli et al. observed no major complications following DEB-TACE or TAE in their study, further supporting the safety profile of these interventions [11].

#### 3.1.3. TARE

##### Outcomes and Complications

TARE has shown promising results in reducing tumor burden and alleviating symptoms in RCC. Preclinical studies in porcine models have demonstrated the safety and efficacy of Y90 TARE in RCC management, achieving localized tumor necrosis and surpassing TAE in tumor control [12]. Several case studies and early clinical trials also support these findings, reporting effective disease control, minimal renal toxicity, and reduced postoperative pain compared to other embolization techniques [12,60]. For instance, Hamoui et al. reported over a year of disease stabilization in a case of metastatic RCC treated with Y90, including pain palliation and decreased hematuria. Similarly, Spyridonidis et al. documented complete tumor necrosis and size reduction in addition to resolved hematuria and significant symptomatic relief in a patient with large, inoperable RCC [61,62].

The RESIRT trial, a Phase I study led by Souza et al., further highlighted TARE’s potential. The trial showed TARE to be well tolerated even at doses of up to 300 Gy, with no significant impact on renal function. Among 21 patients, 4.8% achieved a partial response, 90.5% had stable disease, and 4.8% demonstrated progressive disease. The 12-month OS rate was 76.2%, with a median PFS of 12 months. Additionally, TARE maintained stable QoL scores throughout the year [12].

TARE also demonstrates a favorable safety profile in the management of advanced RCC. Most adverse events were mild to moderate, including fatigue (38.1%), back pain (28.6%), vomiting (28.6%), and decreased appetite (23.8%), all of which were managed with supportive care. While 47.6% of patients experienced serious adverse events over 12 months, these were largely attributed to disease progression or unrelated factors, such as pre-existing conditions or complications like over-anticoagulation. Importantly, no grade ≥3 adverse events were directly associated with TARE, affirming its safety under established protocols [12].

#### 3.1.4. Combination Therapies: TAE–Ablation

##### Outcomes and Complications

The combination of TAE followed by percutaneous ablation offers a promising approach for RCC management. While percutaneous ablation is effective for small renal masses, it has limitations, such as difficulties with tumor visualization during CT-guided procedures and local recurrence due to incomplete tumor coverage [13]. TAE complements ablation by reducing tumor vascularity, enhancing visualization with lipiodol-containing embolic agents, and mitigating the heat-sink effect, a common complication that occurs in ablative therapies where blood flow in highly vascular tumors dissipates thermal energy [63,64].

This combined approach has shown significant efficacy, particularly for tumors smaller than 4 cm. In a pilot study led by Nakasone et al., sequential renal arterial embolization followed by radiofrequency ablation (RFA) in patients with unresectable RCC achieved a technical success rate of 100%. Post-treatment imaging at one week and at three months confirmed complete necrosis in all treated segments, with no local recurrence observed over a mean follow-up of 47 months [13]. Similarly, Arima et al. reported a 97% local tumor control rate at 24 months after ethanol-based embolization and RFA in 36 patients [15]. Yamakado et al. also demonstrated 100% eradication of enhancing tumors one week after treatment in patients with small RCCs [14]. These findings highlight the potential of TAE–ablation combination therapy to enhance outcomes, particularly for central or mixed tumors where ablation alone may be limited by challenges such as the heat-sink effect [17].

The safety profile of TAE combined with ablation has also been favorable, with minimal complications reported. Nakasone et al. observed no major complications, with only mild, self-limiting adverse events such as back pain, fluid collections, subcapsular hematomas, hematuria, and nausea. Renal function remained stable, with no significant changes in serum creatinine or GFR post-treatment [13]. Similarly, Winokur reported no severe complications across multiple studies, noting back or flank pain as the most common adverse event. Rare complications, such as pyonephrosis and abscesses, occurred in only 3% of cases and were effectively managed with antibiotics and drainage. Compared to nephron-sparing surgeries, which have complication rates of up to 30%, the combination of TAE and ablation shows promise as a safer alternative [17].

#### 3.1.5. Combination Therapies: TACE–Systemic Therapies

##### Outcomes and Complications

The combination strategy of TACE with systemic therapy has also emerged as a promising alternative. By combining the localized tumor control of TACE with the systemic benefits of TKIs such as sunitinib, this approach provides a dual mechanism that may mitigate resistance and improve therapeutic outcomes. For instance, in a study conducted by Lu et al., patients in the TACE–sunitinib group demonstrated significantly improved survival metrics compared to sunitinib monotherapy. The objective response rate was notably higher in the combination group (66.0% vs. 39.2%), as was the DCR (85.1% vs. 66.7%). These advantages translated to longer median PFS and OS, with the combination achieving a median PFS of 15.6 months and a median OS of 35.0 months, compared to shorter durations with sunitinib alone [19].

Importantly, the addition of TACE did not significantly increase the incidence of sunitinib-related adverse events, maintaining a safety profile comparable to monotherapy. Both groups reported similar rates of common adverse events associated with sunitinib, such as fatigue, anorexia, hypertension, hand-foot syndrome, diarrhea, and rash. However, TACE was linked to higher rates of PES, with increased occurrences of abdominal pain (55.3% vs. 13.7%), fever (61.7% vs. 7.8%), and vomiting (40.4% vs. 19.6%). These findings indicate the need for careful monitoring and management of localized embolization effects during combination therapy [19].

### 3.2. LRT for the Prostate

#### 3.2.1. Background

PCa is the second most common cancer among men and the eighth leading cause of cancer-related mortality worldwide [48]. Every year, an estimated 1.5 million new cases are diagnosed and over 350,000 deaths are reported globally [48,65]. Its incidence exhibits significant geographic variability, with higher rates observed in developed regions such as North America, Northern Europe, and Australia. This disparity is primarily attributed to the widespread use of prostate-specific antigen (PSA) screening and differences in healthcare access [48]. Notably, more than 80% of men are estimated to develop PCa by the age of 80, a burden expected to increase with aging populations and advancements in diagnostic technologies [65].

The management of PCa is highly individualized, tailored to disease stage and patient-specific factors such as life expectancy and comorbidities. Localized disease may be addressed with AS, radical prostatectomy, or radiation therapy (RT), whereas advanced and metastatic cases typically require androgen deprivation therapy and novel hormonal agents [65,66]. Despite advancements in surgical techniques, such as robotic-assisted radical prostatectomy (RARP), functional complications and significant morbidities associated with surgery remain major concerns [67]. Similarly, palliative interventions like transurethral resection of the prostate (TURP), commonly used to relieve bladder outlet obstruction in metastatic PCa, carry risks including bleeding, incontinence, and sexual dysfunction [20,68]. As such, contraindications and resistance to standard treatment remains a significant challenge, emphasizing the need for innovative approaches [65].

Minimally invasive therapies offer promising alternatives by leveraging the prostate gland’s distinct vascular anatomy for targeted treatments. Techniques such as prostate artery embolization (PAE) and prostate artery chemoembolization (PACE) selectively target the prostatic arterial supply to induce ischemia or deliver localized chemotherapeutic agents. Early studies demonstrated their potential in reducing prostate volume (PV), alleviating lower urinary tract symptoms (LUTS), and achieving tumor regression with minimal systemic side effects, positioning these therapies as significant advancements in PCa management.

#### 3.2.2. TAE

##### Outcomes and Complications

Although PAE was initially developed for managing LUTS in patients with benign prostatic hyperplasia (BPH), emerging evidence suggests that PAE may hold utility in the management of localized and advanced PCa [20,25]. For instance, the application of PAE for palliation in PCa has demonstrated promising results. In a prospective trial of 15 patients with advanced PCa, Malling et al. investigated PAE for managing LUTS and catheter-dependent urinary retention. By employing bilateral PAE using 300–500 µm microspheres and the PErFecTED (proximal embolization first, then embolize distal) technique (Merit Medical Systems, Jordan, Utah, USA), Malling et al. achieved a mean 12.2-point reduction in the International Prostate Symptom Score (IPSS). However, the resolution of urinary retention was limited, with success in only 17% of catheter-dependent patients [20,25].

Parikh et al. further explored the role of PAE in patients with chronic prostatitis/chronic pelvic pain syndrome (CP/CPPS) secondary to RT. RT, while a cornerstone treatment for localized PCa, often leads to chronic genitourinary toxicity, with CP/CPPS frequently occurring in patients receiving high central urethral doses [21]. In their study, bilateral PAE with 300–500 µm Embospheres improved symptoms in 89% of patients, while QoL improved in 78% [21].

PAE has also demonstrated efficacy in managing refractory hematuria associated with advanced PCa. Tapping et al. reported successful cessation of hematuria in all four patients treated with PAE, with only one patient requiring repeat embolization after 13 months due to recurrence. Sustained improvements in PV and IPSS were noted at 12- and 18-month follow-up [23]. Similarly, Chen et al. achieved a clinical success rate of 67% in nine patients, with gross hematuria resolving within one week. Although mild residual bleeding persisted in two cases, PAE proved effective and safe for hematuria management [22,23].

The safety profile of palliative PAE is also favorable. Common adverse events include PES and bladder spasms, typically resolving with symptomatic treatment [20,21]. Malling et al. also reported isolated cases of groin hematoma, balanitis, and urinary tract infection, which were all managed successfully. Importantly, studies by Tapping and Chen reported no severe PAE-related adverse events, underscoring the procedure’s safety [22,23,69].

The primary application of PAE in PCa management has also shown promising results, particularly as an adjunct to AS and as a potential treatment for advanced disease. In a prospective study, Frandon et al. evaluated unilateral PAE in 10 men with low-risk PCa under AS, utilizing 300–500 µm Embospheres targeted to the affected prostatic lobe. The procedure achieved successful embolization in all cases, with imaging confirming partial, heterogeneous ischemia localized to the targeted region. At six months, 40% of patients had negative biopsies in both targeted and systematic assessments, and 30% showed no visible lesions on multiparametric MRI. Additionally, IPSS improved from a median of 5 to 1, with no reported cases of erectile dysfunction or incontinence among the 90% of patients who remained under AS after one year [20,24].

For advanced PCa, Burkhardt et al. investigated PAE with 250–400 µm microspheres in nine men. Significant improvements in urinary outcomes were observed, including a median IPSS reduction of six points and a decrease in post-void residual urine volume from 70 mL to 10 mL within 12 weeks. However, tumor-related outcomes varied. While one patient exhibited significant necrosis (≥50%), most had more than 50% viable tumor on imaging. A slight increase in PV was also noted, suggesting the need for further research to explore PAE’s cytoreductive potential in larger and more diverse populations [25].

The safety profile of PAE as a primary treatment modality has been consistently favorable. Frandon et al. reported minor complications in 30% of patients, including urinary infection and superficial hematoma, both of which were resolved with antibiotics and analgesics. Similarly, Burkhardt et al. noted a single Clavien–Dindo grade I complication, with one patient experiencing transient prostatic pain that resolved within three days with oral analgesics. The absence of major adverse events further emphasizes PAE’s safety and feasibility as a minimally invasive option in PCa management [24,25].

#### 3.2.3. TACE

##### Outcomes and Complications

The use of TACE in the prostate, also known as PACE, has emerged as a minimally invasive alternative in the management of advanced PCa complicated by LUTS. Unlike ablative therapies such as high-intensity focused ultrasound (HIFU) and cryotherapy, which carry a notable risk of metachronous tumor recurrence in residual prostate tissue, PACE offers superior tumor control with a more favorable safety profile by delivering cytotoxic agents directly to the prostate. In addition, recent advancements in PACE with drug-eluting beads (DEB-PACE) have shown promise not only in alleviating symptoms but also in managing tumor burden, highlighting its potential as an innovative strategy for advanced PCa treatment [26,29].

In a study by Wang et al., DEB-PACE was evaluated in 32 patients with advanced PCa complicated by gross hematuria and urinary retention. The procedure utilized epirubicin-eluting 30–60 μm HepaSpheres combined with intra-arterial docetaxel infusion, chosen for its established efficacy in metastatic hormone-resistant prostate cancer [27,29]. The study achieved 100% clinical success in controlling hematuria, with sustained bleeding control in 86.7% of patients over a mean follow-up of 27 months. Additionally, 77.3% of catheter-dependent patients resumed spontaneous voiding within three weeks, and 88.2% remained catheter free throughout the follow-up period [27].

Similarly, Guan et al. evaluated DEB-PACE in eight patients presenting with LUTS or hematuria. Using 70–150 μm Callispheres beads loaded with epirubicin, the study achieved a 100% technical success rate. Significant symptomatic relief was observed within one week of treatment, with catheter removal on average within one week post-procedure. At three months, patients experienced a mean PV reduction of 53.5% and significant improvements in IPSS and QoL [26].

In addition to symptom management, PACE has demonstrated encouraging oncological outcomes. In a proof-of-concept study, Pellerin et al. treated five dogs with biopsy-confirmed PCa using 50–100 µm polymer beads loaded with 18 mg of docetaxel. Results showed a median PV reduction of 47% at 30 days and 78% at 60 days post-treatment. Histopathological analysis revealed extensive necrosis in 88% of the prostate, with only 2–25% viable tumor cells and low mitotic activity [29,69].

Clinical studies have also reinforced PACE’s potential in localized PCa. For instance, Wang et al. reported extensive tumor ischemia and necrosis, with complete response in 73.3% of cases. One-, two-, and three-year survival rates were 96.7%, 86.7%, and 73.3%, respectively, with a mean PV reduction of 55.5% [27]. Guan et al. also reported significant reductions in PSA levels and PV, reinforcing PACE’s potential as a therapeutic option for advanced PCa [26]. In another study, Pisco et al. evaluated 20 men treated with docetaxel-loaded 150–300 µm microspheres. The study achieved an 80% technical success rate and an 81.3% biochemical success rate, defined as PSA <2 ng/mL at one month. Patients with Gleason scores ≥7 experienced tumor volume reductions exceeding 50%, while those with Gleason scores of 6 showed no significant MRI changes. Long-term biochemical success was observed in 62.5% of patients at 12–18 months, with marked decreases in PV and PSA levels. Notably, one patient undergoing radical prostatectomy post-PACE had no detectable malignancy on histopathological examination despite an initial Gleason score of 4 + 5 [28,69].

PACE has also demonstrated a favorable safety profile, with most complications being mild and transient. Pellerin et al. observed no major complications, with no reports of systemic toxicity or non-target embolization, attributing this to the minimal systemic absorption of docetaxel [29]. Wang et al. reported no major adverse events among 32 patients, with minor complications observed in only 12 patients. In addition, no significant chemotherapeutic toxicity was noted, and none of the six patients who reported normal sexual function before TACE experienced erectile dysfunction post-procedure [27]. Guan et al. also observed a low complication rate, with only one patient experiencing a small perineal punctate skin defect, and another developing a minor ischemic area on the penile urethral orifice. Both complications were managed with symptomatic treatment and resolved within two weeks [26].

On the other hand, Pisco et al. observed a 31% complication rate, predominantly involving transient adverse events. Two patients experienced temporary sexual dysfunction that resolved within 10–12 months, and one patient developed acute urinary retention which resolved after one week of catheterization. A single major complication involving bladder wall ischemia required surgical intervention, but the patient’s PSA levels decreased significantly post-procedure, with histopathology showing no residual malignancy [28].

#### 3.2.4. TARE

##### Outcomes and Complications

The use of TARE with Y90 microspheres offers a novel and promising approach for the treatment of PCa [70]. In a preclinical proof-of-concept study, Mouli et al. demonstrated the technical feasibility and safety of TARE in a canine model of prostatic hyperplasia. The study successfully delivered Y90 microspheres to the targeted prostatic hemigland in all 14 animals. Imaging confirmed precise dose localization, with significant dose-dependent reductions in hemigland sizes of up to 60% observed by 40 days post-treatment. Importantly, no extraprostatic radiographic or histologic changes were observed, reinforcing the localized therapeutic effect of TARE [70].

Building on these findings, the ongoing VOYAGER trial is currently evaluating the clinical application of TARE. This interventional study aims to determine the maximum safe dose of the TheraSphere PCa device in patients with clinically localized PCa. Enrolling up to 36 patients, the trial will evaluate the technical feasibility, safety, efficacy, and QoL outcomes over a five-year follow-up period. The results are expected to provide critical insights into dose optimization, long-term oncological outcomes, and the potential role of TARE in PCa management [71].

TARE has shown a favorable safety profile in preclinical studies. For instance, Mouli et al. reported no genitourinary or colorectal toxicities across all dose groups in their canine study. Radioactivity was also undetectable in urine and feces during follow-up, and serum chemistries were stable [30]. Nonetheless, the collateral blood supply of the prostate to adjacent structures, such as the bladder and rectum, necessitates caution. Unlike benign embolic particles, radioactive microspheres pose a higher risk of complications, emphasizing the need for meticulous planning to ensure precise targeting and avoid non-target embolization [16].

### 3.3. LRT for Soft Tissue Sarcomas

#### 3.3.1. Background

STSs are a rare and diverse group of malignancies arising from mesenchymal tissues, including muscles, tendons, fat, blood vessels, and connective tissues [72]. In the United States, the annual incidence of STS is approximately 13,000 cases, with liposarcoma, leiomyosarcoma, and undifferentiated pleomorphic sarcoma being the most frequently diagnosed subtypes in adults [72]. Although STS constitutes less than 1% of all adult cancers, it is notably more prevalent in pediatric populations, accounting for approximately 15% of childhood malignancies [72].

Comprising over 50 distinct histological subtypes, STS exhibits remarkable heterogeneity, with tumors arising in virtually any anatomical site [72]. As such, the prognosis and treatment strategies for STS are strongly influenced by the primary tumor’s anatomical location. The National Comprehensive Cancer Network (NCCN) Guidelines categorize STS into several subtypes for management: STS of the extremities, trunk, or head and neck; retroperitoneal or intra-abdominal STS; desmoid tumors, which infiltrate locally; and rhabdomyosarcomas, the most common subtype in children and adolescents [72,73].

The treatment of STS traditionally relies on a multimodal approach involving surgery, RT, and, in certain cases, systemic chemotherapy [73]. Surgical resection with negative margins remains the cornerstone of therapy for localized STS, with adjuvant RT commonly employed to reduce the risk of local recurrence. However, achieving adequate surgical margins can be particularly challenging in anatomically complex regions, such as the retroperitoneum or head and neck, leading to higher rates of recurrence and treatment-related morbidity [72]. For patients with advanced or unresectable disease, RT or systemic chemotherapy serve as a primary treatment modality. However, systemic chemotherapy has demonstrated limited efficacy in improving survival across most STS subtypes, underscoring the need for novel therapeutic strategies [72].

While surgery remains the standard of care for early-stage STS, many patients present with advanced disease where surgical intervention is no longer viable. In addition, while systemic chemotherapy and ablative therapies offer therapeutic potential, they are often constrained in cases involving large, multifocal tumors or lesions near critical structures [33]. LRTs such as TACE and TAE have emerged as promising alternatives with broader applicability and favorable safety profiles [34]. Early studies indicate these modalities are particularly effective in hypervascular tumors, such as sarcomas and metastatic lesions, as they target tumor vascularity to provide symptom relief and achieve tumor control [31,73].

#### 3.3.2. TAE

##### Outcomes and Complications

The clinical efficacy of TAE in STS management has been demonstrated in multiple studies. One of the earliest investigations, conducted by Nagata et al. in 1998, retrospectively analyzed 38 patients with primary or metastatic STS treated with TAE. Significant tumor necrosis was observed in 79% of cases, and 56% of patients experienced tumor size reduction within three months. The median survival was 18 months, with some patients surviving up to 84 months. Additionally, TAE effectively controlled pain, with 89% of patients reporting relief from painful primary tumors within one week of treatment [31,73,74].

While TAE is generally well tolerated, it is associated with minor complications such as fever and localized pain. Nagata et al. reported transient buttock pain following internal iliac artery embolization, which was manageable with analgesics and resolved within a week [31]. They also documented a case of gluteal muscle necrosis resulting from complete embolization of the internal iliac artery in a patient with pre-existing arteriosclerotic obstruction in the contralateral iliac artery, highlighting the importance of thorough vascular assessment and meticulous procedural planning to minimize risks [31].

#### 3.3.3. TACE

##### Outcomes and Complications

The potential of TACE in treating STS has been highlighted in several clinical studies. In a case report by Filho et al., a patient with a large, recurrent retroperitoneal sarcoma resistant to surgical resection and systemic chemotherapy was successfully treated with DEB-TACE, resulting in substantial tumor shrinkage and a complete metabolic response on PET-CT. In addition, the patient experienced improved QoL without notable toxicity [74].

Larger studies further support these findings. Ni et al. evaluated 10 patients with STS refractory to systemic therapy, reporting a median PFS of 9.5 months and OS of 21 months [33,73,74]. Similarly, Jiang et al. studied 39 patients with unresectable STS and reported significant alleviation of cancer-related pain, a mean OS of 23.7 months, and one-, two-, and three-year OS rates of 71.5%, 45.8%, and 32.5%, respectively [34].

TACE has also demonstrated efficacy in specific STS subtypes, such as desmoid tumors. Kim et al. treated patients with extra-abdominal desmoid tumors ineligible for ablation using doxorubicin-loaded DEB-TACE, achieving a 90% tumor volume reduction rate and partial to near-complete tumor necrosis in most cases. Sustained symptomatic improvement, including marked pain relief, was also observed [35]. Similarly, Elnekave et al. reported a median tumor volume reduction of 59% and stable disease in 52% of patients treated for aggressive desmoid fibromatoses, further supporting TACE’s efficacy in this challenging variant [36].

Complications associated with TACE are generally mild and manageable. PES is the most commonly reported complication, presenting with symptoms such as localized pain, fever, nausea, vomiting, and fatigue. Ni et al. observed localized pain in 80% of patients and fever in 70%, with symptoms typically resolving within a few days [33,34,35]. Notably, no serious adverse events or procedure-related mortalities have been reported. Routine post-treatment blood tests in Ni et al.’s study revealed no significant thrombocytopenia, hepatic or renal failure, or grade 3/4 toxicities [33]. Similarly, Jiang et al. reported no major complications or TACE-related deaths, although procedural challenges, such as catheter placement failures, occurred in 23% of cases [34].

### 3.4. LRT for the Pancreas

#### 3.4.1. Background

Pancreatic cancer is one of the most aggressive and fatal malignancies, with a five-year survival rate of less than 10% and an overall survival rate of only 6% [75]. It ranks as the sixth leading cause of cancer-related mortality globally, with over 500,000 new cases and an expected 467,000 deaths annually [48]. The poor prognosis largely stems from late-stage diagnosis, rapid disease progression, and limited therapeutic options [75]. Indeed, the pancreas’ deep retroperitoneal location, coupled with the absence of early, specific symptoms, often results in advanced or metastatic disease at presentation [75,76].

The management of pancreatic cancer is multifaceted, often requiring a combination of surgical, systemic, and radiotherapeutic approaches tailored to the stage and progression of the disease. For patients with localized disease, surgical resection remains the sole curative option. However, proximity to vital vascular structures, such as the superior mesenteric artery, celiac axis, and portal vein, often limits surgical feasibility [75]. When tumors are confined to the pancreatic head, pancreaticoduodenectomy (Whipple procedure) is the preferred intervention, while tumors in the body or tail necessitate distal pancreatectomy, often with concurrent splenectomy [75]. In unresectable locally advanced disease, neoadjuvant chemotherapy with or without radiation is commonly employed to downstage tumors and enhance resectability. Nonetheless, many patients fail to undergo adjuvant chemotherapy following surgery due to postoperative complications or poor performance status, leading to a growing trend of neoadjuvant-first approaches to optimize survival outcomes [75].

Given the rising incidence of pancreatic cancer, developing novel therapeutic strategies has become imperative. LRTs, which bypass systemic barriers by utilizing arterial vasculature to deliver treatment directly to tumor sites, have emerged as a promising option in addressing the challenges of this devastating disease [76]. While the application of LRTs in pancreatic cancer has been relatively limited due to the potential risk of complications such as acute ischemic pancreatitis, recent advancements, such as superselective embolization techniques, have been developed to enhance drug delivery while minimizing damage to the pancreas’ critical exocrine and endocrine functions [37]. Furthermore, approaches such as intra-arterial (IA) chemotherapy infusion and transarterial microperfusion (TAMP) have shown promise in improving therapeutic outcomes while reducing systemic toxicities [38,41,77].

#### 3.4.2. TAE

##### Outcomes and Complications

TAE has shown promise in selectively targeting pancreatic tumors while minimizing systemic exposure. In a recent animal study, Cline et al. investigated the safety and efficacy of particle-based TAE in a porcine model. Using common femoral artery access, the study selectively embolized the dorsal pancreatic artery with 100–300 μm microspheres in 14 Yorkshire pigs. All animals survived the 14-day study period, demonstrating a return to baseline food and water intake by post-procedural day 1. On pathologic analysis, approximately 25% of the pancreas was found to be ischemic, with fibrosis observed in the distal body and tail. Histologic evaluation revealed the loss of both acinar and islet cells within the embolized region, without evidence of active inflammation. Notably, the inflammation remained confined to the targeted area, with no extension into adjacent pancreatic tissue [37].

Based on these findings, Cline et al. suggested that the mechanism of particle embolization, which prevents collateral blood flow by acting distally, may reduce the risk of pancreatitis. This approach potentially avoids the rapid restoration of oxygenation to ischemic tissue, a key factor in reperfusion injury commonly implicated in the pathogenesis of pancreatitis [37].

No complications were reported in the study, with no evidence of distress or clinical signs of pancreatitis. Although pancreatic enzyme levels, including amylase and lipase, exhibited significant increases at 24 and 48 h post-embolization, they normalized at the study’s conclusion. In addition, endocrine and exocrine functions remained largely intact, as indicated by normal glucose tolerance test results and adequate weight gain throughout the study period [37].

#### 3.4.3. Intra-Arterial (IA) Therapies

##### Outcomes and Complications

IA chemotherapy has emerged as an innovative strategy in the management of pancreatic cancer by enhancing the delivery of therapeutic drugs directly to tumors. Regional IA chemotherapy (RIAC) has demonstrated particular promise. By optimizing local drug delivery, RIAC leverages the dose-dependent nature of chemotherapy efficacy while minimizing systemic exposure to enhance treatment outcomes [78]. In a systematic review comparing RIAC to systemic chemotherapy in pancreatic cancer, Cao et al. reported that RIAC achieved superior partial remission rates and lower complication rates. Additionally, RIAC resulted in longer median OS, ranging from 10 to 21 months in RIAC groups compared to 4.8 to 14 months in systemic chemotherapy groups [38]. A recent Phase II clinical trial comparing IA and IV infusion of gemcitabine and oxaliplatin (GEMOX) further demonstrated that the 6-month OS was higher in the IA group (100% vs. 84%), with fewer adverse effects than the IV group [39].

Innovative approaches to enhance IA chemotherapy have also emerged. One strategy involves embolizing pancreatic arteries originating from the superior mesenteric artery to achieve a single blood supply to the tumor. For example, in a study of 20 patients, Tanaka et al. found that IA infusion of 5-fluorouracil (5-FU) combined with intravenous gemcitabine following superselective coil embolization resulted in a tumor response rate of 68.8%, a median OS of 9.8 months, and PFS of 6.0 months [40]. Another promising technique, transarterial microperfusion (TAMP) therapy, uses a targeted IA delivery catheter and has shown encouraging early-phase results, with a median OS of 12.5 months [41]. Further innovations include the TIGeR-PaC trial (NCT03257033), a Phase III study currently evaluating the efficacy of local double-balloon-mediated delivery of IA gemcitabine compared to IV gemcitabine/nab-paclitaxel in patients with locally advanced pancreatic cancer [79].

Nonetheless, IA therapies are not without complications. Minor toxicities associated with IA infusion include anemia, thrombocytopenia, and peripheral neuropathy, while IA 5-FU combined with IV gemcitabine has been linked to neutropenia, constipation, and catheter dislocation [39,40]. Gastrointestinal symptoms such as nausea and emesis are frequently observed with TAMP therapy [41].

Major adverse events, although less common, have also been reported. Tanaka et al. observed severe neutropenia and thrombocytopenia with IA 5-FU administration in their study, while sepsis was noted in 17.3% of patients undergoing TAMP therapy [40,41].

### 3.5. LRT for the Brain

#### 3.5.1. Background

GBM is the most common primary brain tumor in adults, accounting for over 45% of all malignant primary brain and central nervous system (CNS) tumors [80]. The annual incidence is estimated at 3.2 per 100,000 people, with a peak occurrence in individuals aged 64 [80,81]. Despite advancements in multimodal therapies, survival rates remain dismal, with a median survival of approximately 15 months and a five-year survival rate of less than 10% [82]. As the U.S. population continues to age, the incidence of GBM is expected to rise, highlighting the importance of advancing therapeutic approaches [80].

The standard of care for GBM involves maximal safe surgical resection followed by RT and temozolomide chemotherapy, commonly referred to as the “Stupp protocol” [42,80]. Surgical resection provides multiple benefits, including cytoreduction, definitive histological diagnosis, and tumor genotyping. Achieving gross total resection significantly improves survival outcomes, with a 60% increase in one-year survival and a 20% improvement in two-year survival compared to subtotal resection [80]. Advanced RT options, such as stereotactic radiosurgery, brachytherapy, and proton beam therapy, complement surgical approaches. Tumor-treating fields have also emerged as a guideline-recommended adjunct due to their demonstrated efficacy in prolonging survival [80].

Despite these advances, the highly invasive growth patterns of GBM pose a significant challenge to effective management. The tumor’s molecular landscape, which is characterized by extensive genetic and epigenetic alterations, promotes abnormal proliferation, angiogenesis, and treatment resistance [80]. GBM cells also employ immune evasion mechanisms within the immune-privileged CNS, which lacks antigen-presenting cells and functional lymphatics [80]. GBM’s resistance to conventional therapies is further compounded by the tumor’s proximity to critical brain structures, which restricts the extent of surgical and radiological interventions [80]. Tumor cells also migrate along white matter tracts, blood vessels, and the subependymal zone, infiltrating areas beyond the resection margin. This invasive behavior makes complete surgical resection virtually impossible and leaves behind residual tumor cells that contribute to recurrence [82,83].

Another major obstacle in GBM treatment is the blood–brain barrier (BBB), a highly selective, semi-permeable membrane that regulates the passage of substances between the bloodstream and the CNS [84]. The BBB limits the penetration of many systemic therapies, including most chemotherapeutic agents and biologic treatments, into the tumor microenvironment. In addition, the BBB is often disrupted in regions of high tumor vascularity, further contributing to suboptimal drug delivery and therapeutic resistance [84].

Endovascular therapies, including embolization and IA drug delivery, represent innovative strategies in GBM management by addressing the challenges posed by the BBB. These LRTs enable the targeted delivery of therapeutic agents directly into the cerebral arteries supplying the tumor, bypassing the BBB to achieve high intra-tumoral therapeutic concentrations while minimizing systemic and cerebral toxicities [85]. While ischemic damage to healthy brain tissue from embolic agents remains a concern, advancements in catheter technology and embolic agent design have significantly enhanced the precision, safety, and efficacy of these techniques, making them a promising approach for improving GBM outcomes [26,42,86].

#### 3.5.2. TARE

##### Outcomes and Complications

Y90 TARE represents a novel approach to GBM treatment, with preclinical studies and emerging human trials demonstrating its feasibility, safety, and potential efficacy [87,88]. Pasciak et al. evaluated Y90 TARE in a canine model of spontaneous intra-axial brain tumors, achieving absorbed tumor doses of 45.4–64.1 Gy while limiting healthy brain exposure to 15.4–33.3 Gy. Tumor volume reductions ranged from 24% to 94% one month post-treatment, with partial responses observed in 60% of cases at six months [18,42,89].

Building on these findings, the ongoing FRONTIER trial (NCT05303467) is investigating the application of Y90 TARE in patients with recurrent GBM. With absorbed doses of 40 Gy targeted to tumor volumes, this first-in-human study focuses on the safety and efficacy of selective IA Y90 microsphere delivery. Key endpoints include dose-limiting toxicities, response rates, PFS, and OS. The trial also incorporates advanced imaging modalities, such as Y90 PET and MRI, to enhance understanding of dosimetry and dose distributions [43].

Nonetheless, complications remain an inherent risk in endovascular embolization, particularly for brain tumors. General risks include embolic material reflux, vessel rupture, and ischemic events, which can result in hemorrhage or ischemia. For instance, Pasciak et al. reported transient neurological deficits in six dogs, although histopathological analyses revealed no significant brain atrophy and only rare chronic infarcts. They also reported a case of unexpected mortality, attributed to a hypervascular tumor occupying substantial cerebral volume. Factors such as outdated pretreatment imaging, prolonged angiography, and overhydration from high intravenous fluid rates likely compounded procedural risks. Although histopathological analysis revealed no widespread ischemia, their study demonstrates the importance of optimized patient selection, real-time imaging, and comprehensive post-procedural monitoring in minimizing complications [42].

#### 3.5.3. IA Therapies

##### Outcomes and Complications

IA therapies offer an innovative approach in enhancing local drug delivery for GBM while minimizing systemic toxicity. Due to non-selective drug infusion through the carotid or vertebral arteries, early studies investigating IA therapies were marked by significant neurotoxicity [85]. However, recent advancements in catheter technology, such as microcatheters and microwires, have transformed IA therapy into a highly localized treatment [16,84]. For instance, techniques such as selective intra-arterial cerebral infusion (SIACI) enable targeted delivery of therapeutic agents and confine the volume of distribution to the tumor [18,84]. Enhanced imaging technologies have also improved angiographic precision, further refining tumor localization and IA therapy delivery [90].

Nonetheless, investigations into IA therapies for GBM have produced mixed results. Early studies suggest that IA delivery achieves significantly higher local drug concentrations compared to IV methods, with reported increases of up to 300-fold in tumor drug concentrations and an overall reduction in systemic toxicities [84,91]. Nonetheless, while IA therapy has shown some improvement in time to progression (TTP), OS differences between IA and IV approaches have been statistically insignificant [84,85].

Recent studies have focused on the use of specific chemotherapeutic agents in conjunction with BBB-disrupting medications to enhance drug delivery. Patel et al. demonstrated the feasibility and safety of IA bevacizumab (15 mg/kg) with osmotic BBB disruption in 23 patients with newly diagnosed GBM. Administered up to three times post-resection with concurrent chemoradiation, this approach achieved a mean OS of 23.1 months without life-threatening adverse events [47,90,92]. IA therapy has also been explored in enhancing therapies which have historically demonstrated limited efficacy in GBM. For instance, a Phase I trial of SIACI with cetuximab, an EGFR inhibitor, established a maximum tolerated dose of 250 mg/m^2^ without significant adverse events, dose-limiting toxicities, or neurological deficits [46,90].

Combination therapies further highlight the potential of IA therapeutic approaches. A study combining IA bevacizumab and cetuximab with BBB disruption in 13 children with high-grade glioma or diffuse intrinsic pontine glioma (DIPG) yielded promising results, with 92% of patients showing reduced tumor enhancement on day 1 post-treatment MRI and no dose-limiting toxicities. Several patients with DIPG also achieved partial or complete responses, with a mean OS of 17.3 months [45,90].

Complications associated with IA therapy are typically mild and manageable. Patel et al. reported predominantly grade 1 or 2 adverse events following SIACI of bevacizumab, most of which were resolved with minimal intervention. Among the 12 grade 3 events, neurologic complications, such as seizures and aphasia, were effectively treated [44]. Similarly, McCrea et al. reported stable neurological examinations and no significant complications or MRI evidence of hemorrhage or stroke in their study. Rare minor events, such as mild rashes and occasional epistaxis, were self-limiting [45].

Serious adverse events, however, have been documented. Chakraborty et al. reported two serious adverse events during IA cetuximab infusions: an anaphylactic reaction requiring procedural cessation, and a case of cerebral edema with a small tumor-bed hemorrhage. Both were successfully managed with supportive care, and subsequent protocol adjustments, such as premedication with antihistamines, prevented recurrence [46].

## 4. Challenges and Future Directions of Extrahepatic LRTs

While LRTs have shown promise in the management of extrahepatic malignancies, significant challenges remain in their clinical application. For instance, a prevalent barrier across the extrahepatic application of TAE, TACE, and TARE is the limited strength of existing evidence. Many studies are retrospective in nature, characterized by small sample sizes and short follow-up durations, which limit the ability to draw definitive conclusions regarding efficacy and long-term outcomes [10,11,12]. In addition, procedural variability, ranging from the selection of embolic agents to the targeting precision of tumors, introduces inconsistencies that complicate protocol standardization and reproducibility across clinical settings [22,23].

The discrepancy between imaging-based assessments and actual histopathological outcomes is another major challenge. For instance, in the employment of TACE for RCC, viable tumor cells are often detected histologically despite radiographic indications of complete necrosis, raising concerns about the cytoreductive efficacy of the procedure [10,11]. Similar limitations are observed in PCa embolization, where the lack of post-procedure histopathological confirmation weakens conclusions regarding tumor response [24,25]. Studies evaluating TARE face similar barriers, such as microdosimetry variability and the challenge of achieving uniform dose distribution, which impact treatment predictability and reproducibility [30].

While TAE, TACE, and TARE have demonstrated potential benefits, their precise role within broader oncologic treatment paradigms remains uncertain. For example, combination strategies such as TAE with ablation and TACE with systemic immunotherapy for RCC have shown preliminary efficacy, but the absence of well-powered, prospective trials limits their integration into routine clinical practice [19,93,94]. Similarly, the employment of PAE in palliative settings is challenged by the heterogeneity of patient populations, varying treatment endpoints, and inconsistent definitions of clinical success, making it difficult to establish standardized protocols [20,22].

Future research efforts should prioritize large, multicenter randomized controlled trials to validate the efficacy and safety of LRTs in diverse patient populations. Standardizing procedural techniques, including optimizing embolization parameters and refining patient selection criteria, will be essential in improving treatment consistency and outcomes. Innovations such as nanoparticle-based embolization, liquid embolics for enhanced drug delivery, and precision-guided dosimetry in TARE may help overcome current limitations and enhance therapeutic efficacy [47,95,96]. In addition, the integration of LRTs into multimodal treatment strategies, such as approaches combining immune checkpoint inhibitors or RT, represents a promising avenue for expanding their clinical applicability [36,93]. The ongoing VOYAGER trial for TARE in PCa and the feasibility studies exploring IA chemotherapeutic infusion for GBM underscore the need for continued translational research to refine these approaches and establish evidence-based guidelines for their implementation [70,97].

Ultimately, addressing these challenges through rigorous clinical investigations and technological advancements will be pivotal in solidifying the role of LRTs in the evolving landscape of oncologic care. By enhancing precision, refining patient selection, and integrating LRTs into comprehensive treatment frameworks, these modalities have the potential to significantly improve outcomes for patients with extrahepatic malignancies.

## 5. Conclusions

LRTs such as TAE, TACE, and TARE are gaining increasing recognition for their expanding role beyond hepatic malignancies. This review highlights their evolving applications in challenging cancers such as RCC, PCa, STS, pancreatic cancer, and GBM. The precision and minimally invasive nature of these therapies make them particularly compelling for patients with limited treatment options due to comorbidities or poor surgical candidacy. By delivering targeted treatment directly to tumor vasculature with minimal systemic toxicity, LRTs show promise in achieving tumor control, enhancing patient outcomes, and improving healthcare efficiency by reducing resource utilization and costs.

Despite these advances, significant gaps remain. Due to small study sizes and variability in design, as well as the lack of long-term outcome data, further research is needed. Future efforts should focus on refining patient selection criteria, optimizing procedural protocols, and exploring therapeutic combinations. By addressing these challenges and building on existing successes, LRTs are poised to redefine cancer management paradigms for extrahepatic cancers.

## Figures and Tables

**Table 1:** Outcomes of locoregional therapies in extrahepatic malignancies.

Cancer type	LRT	Indication	Outcomes	Complications	Key Insights
**Renal Cell Carcinoma (RCC)**	TACE	Palliative, potential for treating localized RCC in patients ineligible for surgery	Median PFS: 21.4 mo [10] Median OS: 24.6 mo [10] Tumor Necrosis: TACE (88.3%) vs. TAE (29.4%); histopathological analysis confirmed necrosis rates for TACE (87.5%) vs. TAE (26%) [11]	Minor adverse events: Abdominal pain (34.3%); nausea or vomiting (14.3%); abdominal distension (11.4%); moderate fever (8.6%); hematuria (2.9%) [10] Major adverse events: None [10,11]	DEB-TACE with doxorubicin-loaded beads is a safe, effective, and feasible palliative treatment for unresectable RCC [10]. It also offers a superior cytoreductive effect compared to TAE while maintaining a favorable safety profile for localized RCC [11].
	TARE	Palliative, potential for treating advanced RCC	Median PFS: 12 mo [12] 12 mo OS rate: 76.2% [12] Tumor response: Partial response (4.8%); stable disease (90.5%); progressive disease (4.8%) [12] QoL: Stable QoL scores maintained over 12 mo [12]	Minor adverse events: Fatigue (38.1%); back pain (28.6%); vomiting (28.6%); decreased appetite (23.8%) [12] Major adverse events: 47.6% of patients, but primarily linked to disease progression or TARE-unrelated factors primarily linked; no grade ≥3 adverse events directly attributed to TARE [12]	TARE with Y90 is well tolerated and serves as a feasible strategy for advanced RCC and may be a viable option for patients unsuitable for conventional therapies. Further clinical investigation is warranted [12].
	TAE + Ablation	Potential for small, localized RCC	Technical success: 100% [13] Tumor necrosis: 100% in embolized segments [13]; 75% after one RFA session, 17% after two, 8% after four [14]; 100% after two RFA sessions [15] Tumor size reduction: From 5.2 ± 1.7 cm to 3.6 ± 1.4 cm [14] Local tumor control: 100% during follow-up [16]; 100% in T1N0M0 tumors [15] Recurrence rates: 2.8% overall; 0% in tumors < 4 cm [15]	Minor adverse events: Back/flank pain (50–100%) [13,17]; subcapsular hematoma (6%) [15]/(9%) [14]; fever (27%) [14]; microscopic hematuria (100%) [14]; nausea (3%) [15] Major adverse events: Abscess in the ablated tumor (9%) [14]; pyonephrosis (3%) [15]; retroperitoneal hemorrhage (3%) [15]	The combination of sequential embolization and percutaneous RFA is feasible, relatively safe, and serves as a promising treatment option for inoperable RCC, particularly for tumors smaller than 4 cm [13,14,18].
	TACE + Systemic Therapy	Potential for unresectable or metastatic RCC	Technical success: 100% (TACE + sunitinib group) [19] PFS: 5.6 mo (TACE + sunitinib) vs. 10.9 mo (sunitinib alone) [19] OS: 35.0 mo (TACE + sunitinib group) vs. 25.7 mo (sunitinib alone) [19] Overall response rate (ORR): 66.0% (TACE + sunitinib) vs. 39.2% (sunitinib alone) [19] Disease control rate (DCR): 85.1% (TACE + sunitinib) vs. 66.7% (sunitinib alone) [19]	Minor adverse events: Abdominal pain (55.3%); fever (61.7%); vomiting (40.4%); fatigue (57.4%); decreased appetite (44.7%); hypertension (38.3%); hand-foot syndrome (42.6%); diarrhea (29.8%); rash (17.0%) [19]	TACE combined with sunitinib is a safe and effective complementary treatment for advanced RCC and demonstrates enhancing therapeutic outcomes through synergistic effects [19].
**Prostate Cancer (PCa)**	TAE	Palliative	Technical success: 67% [20], 78% [21], 89% [22], 100% [23] Clinical success: Lower urinary tract symptoms (LUTS) improvement with a mean 12.2-point IPSS reduction [20]; 89% QoL improvement [21]; gross hematuria resolution 67% [22]/100% at 3 mo [23]	Minor adverse events: PES (27%); groin hematoma; self-limiting balanitis; bladder spasms (22%) [20,21] Major adverse events: Urinary tract infection requiring antibiotics; prolonged pelvic pain [20]	PAE is a safe and effective palliative option for managing LUTS, chronic prostatitis/chronic pelvic pain syndrome (CP/CPPS), and hematuria in PCa patients, with potential to improve QoL and manage bleeding [20,21,22,23]
		Potential as an adjunctive therapy with active surveillance (AS) and as treatment for advanced disease	Technical success: 100% [24] Tumor necrosis: ≥50% necrosis in 11% of patients [25] Symptom improvement: Median IPSS reduction by 6 points [25]; no significant changes in IPSS at 6 months [24] PV and tumor volume (TV): PV increased from 19.7 mL to 23.4 mL [25]; TV increased from 6.4 mL to 8.1 mL at 12 weeks [25]	Minor adverse events: Prostatic pain (30%) [24]/(11%) [25]; urinary infection (10%) [24]; superficial hematoma at the puncture site (10%) [24] Major adverse events: None	PAE is a feasible and safe option for PCa patients under AS or with advanced disease, demonstrating potential for functional improvements and cytoreductive effects. Further studies are needed to validate these findings [24,25].
	TACE	Palliative	Technical success: 100% [26,27] Clinical success: Hematuria control (100%) [26,27]; sustained bleeding control (86.7% over 27 mo) [27] spontaneous voiding in catheter-dependent patients (77.3%) [27]; catheter-free rate (88.2%) [27] Mean IPSS reduction: 21.13 points at 1 mo, 24.5 points at 3 mo [26] Tumor response: Complete response in 73.3% [27] PV reduction: 55.5% [27]; 30.28 cm^3^ at 1 mo/46.14 cm^3^ at 3 mo [26] Survival rates: 1 yr (96.7%), 2 yr (86.7%), 3 yr (73.3%) [27]	Minor adverse events: Fever (100%) [27]; nausea (100%) [27]; poor appetite [27]; urethral burning and perineal pain (18.8%) [27]; mild diarrhea with rectal bleeding (6.3%) [27]; perineal punctate skin defect (12.5%) [26]; ischemic area on penile urethral orifice (12.5%) [26] Major adverse events: None	TACE shows promise in managing locally advanced PCa with urinary obstruction or hematuria, though its efficacy and safety require validation in larger prospective studies [26,27].
		Potential for treatment of localized PCa	Technical success: 80% [28] Tumor necrosis: 88% of the prostate with 2–25% viable tumor cells [29] PV reduction: 47% at 30 days/78% at 60 days [29]; 50% PV reduction in patients with Gleason scores ≥ 7 [28]	Minor adverse events: Acute urinary retention (5%); urinary urgency (5%); sexual dysfunction (10%) [28] Major adverse events: Small area of bladder wall ischemia (5%) [28]	TACE shows promise as a feasible and safe treatment for PCa, demonstrating biochemical responses in localized PCa and potential applicability to selected patients [28,29].
	TARE	Potential for treatment of unresectable PCa	Technical success: 100% [30] Tumor response: Dose-dependent hemigland size reductions of up to 60% by 40 days post-treatment [30]	Minor adverse events: None Major adverse events: None	Prostate Y90 radioembolization is safe and feasible, achieving localized dose-dependent changes in the targeted gland without extraprostatic effects. Further investigation is encouraged to support the use of TARE in the management of unresectable PCa [30].
**Soft Tissue Sarcoma (STS)**	TAE	Palliative	Technical success: 100% [31] Clinical success: Pain control 79% [31] Tumor necrosis: Low-density necrosis in 76% of tumors [31] Tumor size reduction: 56% of tumors showed size reduction at 3 mo [31] OS: 18 mo for primary tumors, 12 mo for metastatic [32]; 1 yr OS for primary (38.1%), 1 yr OS for metastatic (38.9%) [31]	Minor adverse events: Local pain (35%); transient local pain and numbness; buttock pain; fever [31] Major adverse events: Gluteal muscle necrosis (2.4%) [31]	TAE is effective for pain and local tumor control in the management of STS [31].
	TACE	Palliative, potential for advanced and refractory STS	Technical success: 100% [33,34,35] Response Rates: Complete response (0%) [33]; partial response (30%) [33]/(39%) [36]; stable disease (40%) [33]/(52%) [36]; progressive disease (30%) [33]/(9%) [36] Symptom improvement: Reduction in pain scores (2.6) [35]; pain control noted with increased relapse intervals [34] Tumor necrosis: Partial to near-complete necrosis (36.4%) [35]; TV reduction: median reduction of 59% [36]; 38.1% at 1 mo/16.5% at further follow-up [35] OS: 21 mo [33]/23.7 mo [34]; 1-year OS (90%) [33]/(71.5%) [34]; 2-year OS (30%) [33]/(45.8%) [34]; 3-year OS (32.5%) [34] DCR: 70% [33]	Minor Adverse Events: Pain (80%) [33]/(54%) [36]/(45.4%) [35]; fever (70%) [33]/(100%) [34]; fatigue (30%) [33]; vomiting (20%) [33]/(59%) [34]; skin toxicity (42%) [36]; re-opening of previous wounds (13%) [36] Major Adverse Events: Vascular spinal cord injury during cannulation [36]	DEB-TACE is a safe and effective treatment for advanced and refractory STS, offering pain relief and improved survival outcomes [33,34,35].
**Pancreatic Cancer**	TAE	Potential for management in pancreatic cancer	Technical success: 100% [37] OS: 100% at 2 weeks [37] Pathologic Findings: Fibrosis in embolized regions without active inflammation in treated or adjacent pancreatic tissue [37] Biochemical Response: Significant increases in amylase and lipase at 24–48 h, normalizing by 2 wks; minimal changes in glucose tolerance at 2 wks [37]	Minor Adverse Events: None Major Adverse Events: None	TAE of the pancreas is feasible and well tolerated in a porcine model, with transient enzyme changes and no significant impact on pancreatic function [37].
	IA Therapies	Palliative, potential for management of advanced or unresectable pancreatic cancer	OS: Regional IA chemotherapy (RIAC) group (10–21 mo) vs. systemic chemotherapy group (4.8–14 mo) [38]; IA group (9.93 mo) vs. IV group (10 mo)/6 mo OS in IA group (100%) vs. IV (83.67%)/1 yr OS in IA group (44%) vs. IV group (20.92%) [39]; IA 5-FU with IV gemcitabine (9.8 mo) [40]; Transarterial microperfusion (TAMP) therapy (12.5 mo) [41] PFS: IA group (5 mo) vs. IV group (4 mo) [39] IA 5-FU with IV gemcitabine (6 mo) [40] ORR: IA group (11.54%) vs. IV group (4%) [39]; IA 5-FU with IV gemcitabine (68.8%) [40] DCR: IA group (53.85%) vs. IV group (44%) [39]	Minor adverse events: IA infusion: Anemia (42.31%); thrombocytopenia (26.92%); neutropenia (11.54%); elevated ALT/AST (23.08%); nausea/vomiting (7.69%); peripheral neuropathy (69.23%); fatigue (7.69%); hyperglycemia (11.54%) [39] IA 5-FU with IV gemcitabine: neutropenia (36.8%); anemia (26.3%); thrombocytopenia (26.3%); constipation (26.3%); abdominal pain (5.3%); stomatitis (5.3%); catheter dislocation (10%) [40] TAMP therapy: Nausea (39%); emesis (35%); abdominal pain (30%) [41] Major Adverse Events: IA infusion: Anemia (7.69%), thrombocytopenia (11.54%) [39] IA 5-FU with IV gemcitabine: Neutropenia (15.8%), thrombocytopenia (5.3%) [40] TAMP therapy: Sepsis (17.3%) [41]	IA therapies, including RIAC and TAMP, offer a favorable safety profile and promising outcomes for locally advanced or unresectable pancreatic cancer [38,39,40,41].
**Glioblastoma**	TARE	Potential for treatment of recurrent GBM	TV Reduction: 24–94% reduction in mass volume at 1 mo in canine study [42]	Minor Adverse Events: Acute neurologic deficits in 75% of animals [42] Major Adverse Events: None	Pasciak’s study demonstrates the feasibility and safety of Y90 TARE in a canine model of GBM [42]. The ongoing FRONTIER trial (NCT05303467) is currently evaluating the safety and efficacy of Y90 TARE in patients with recurrent GBM, with endpoints including dose-limiting toxicities, response rates, PFS, and OS [43].
	IA Therapies	Potential for GBM treatment	PFS: 6, 12, 24, and 60 mo PFS rates of 91.3%, 47.4%, 32.5%, and 5.4% [44] OS: 23.1 mo, with 12, 24, and 36 mo OS rates of 77.3%, 45.0%, and 32.1% [44]; mean OS 17.3 mo with mean post-treatment survival of 7.2 mo [45] Tumor Response: 92% decreased enhancement on day 1 MRI; 50% stable disease and 40% progressive disease at 1 mo MRI [45]	Minor Adverse Events: Epistaxis (20%) and rash (20%) [45]; mild rashes (13.3%) [46]; miscellaneous grade 1 or 2 complications including general symptoms (39.1%), neurologic symptoms (39.1%), gastrointestinal symptoms (21.7%), integumentary symptoms (8.7%), musculoskeletal symptoms (4.3%), metabolic symptoms (8.7%) [44] Major Adverse Events: Seizures (17.4%) [44]; speech-related symptoms (4.3%) [44]; DVT/PE (17.4%) [44]; anaphylactic reaction (6.7%) [46]; cerebral edema with tumor-bed hemorrhage (6.7%) [46]	Repeated IA bevacizumab and IA cetuximab have demonstrated safety and tolerability in GBM, with ongoing trials exploring efficacy for novel strategies like temsirolimus and IA MSCs with oncolytic viruses [44,46,47].

## Data Availability

No new data were created or analyzed in this study. Data sharing is not applicable to this article.
